# Supplementary material for: Synthesis, Cytotoxic Evaluation, and Structure-Activity Relationship of Substituted Quinazolinones as Cyclin-Dependent Kinase 9 Inhibitors
Source: Molecules. 2022 Dec 23;28(1):120. doi: 10.3390/molecules28010120 (PMC9822073; doi:10.3390/molecules28010120)

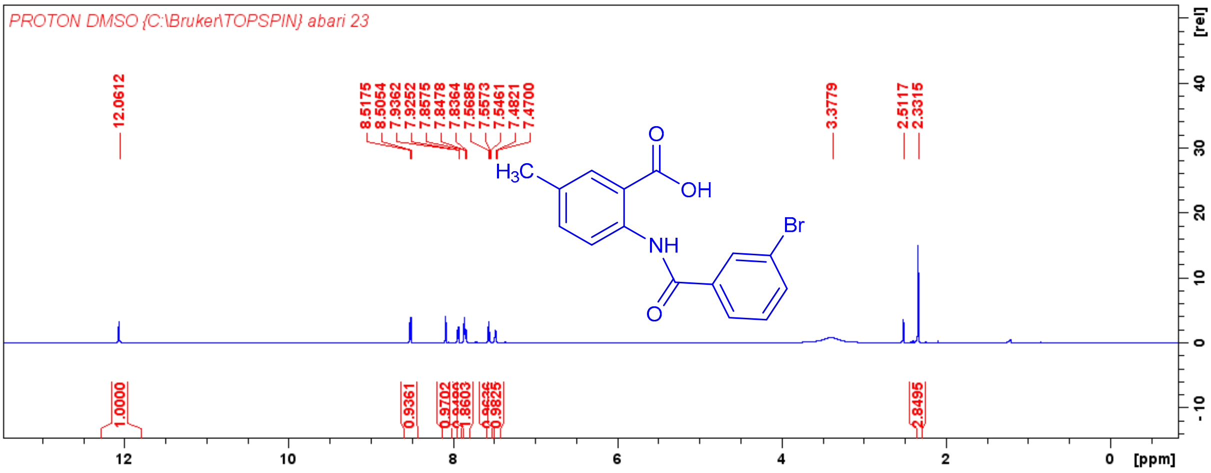
PROTON DMSO {C:\Bruker\TOPSPIN} abari 23
12.0612
8.5175
8.5054
7.9362
7.9252
7.8575
7.8478
7.8364
7.5685
7.5573
7.5461
7.4821
7.4700
3.3779
2.5117
2.3315
H3C
O
OH
NH
Br
O
1.0000
0.9361
0.9702
1.8603
0.9825
2.8495
[rel]
[ppm]

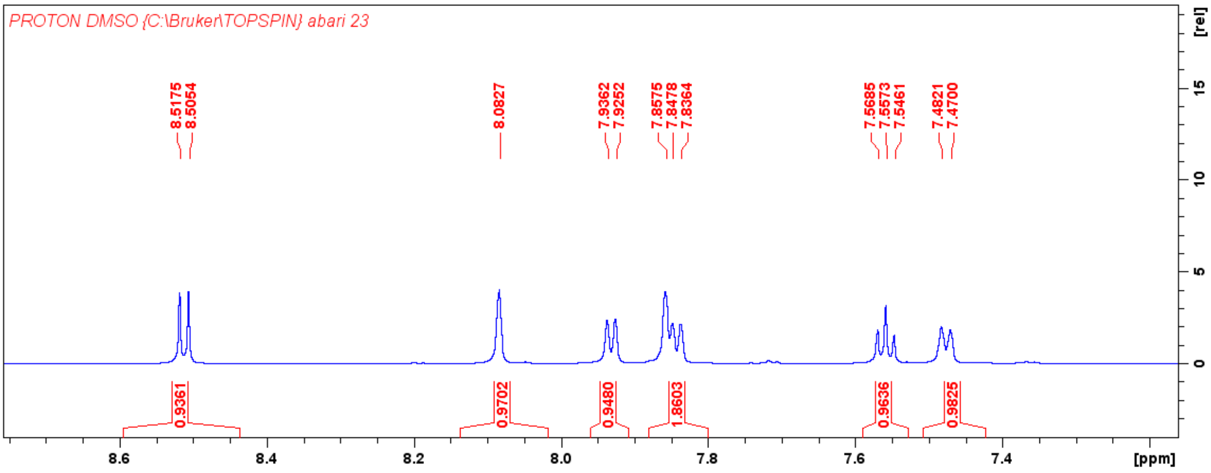
PROTON DMSO {C:\Bruker\TOPSPIN} abari 23
8.5175
8.5054
8.0827
7.9362
7.9252
7.8575
7.8478
7.8364
7.5685
7.5573
7.5461
7.4821
7.4700
0.9361
0.9702
0.9480
1.8603
0.9636
0.9825
[rel]
[ppm]

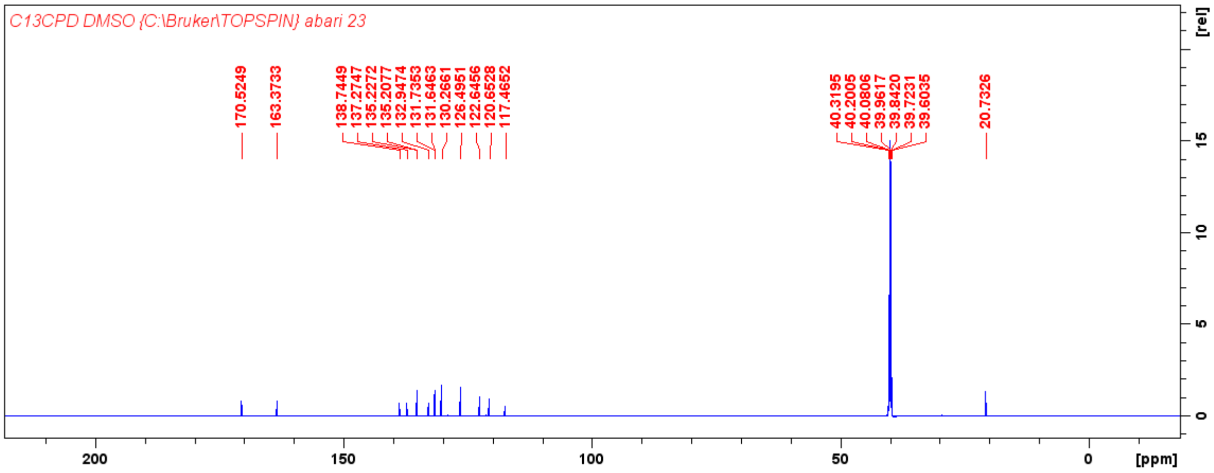
C13CPD DMSO {C:\Bruker\TOPSPIN} abari 23
170.5249
163.3733
138.7449
137.2747
135.2272
135.2077
132.9474
131.7353
131.6463
130.2661
126.4951
122.6456
120.6528
117.4652
40.3195
40.2005
40.0806
39.9617
39.8420
39.7231
39.6035
20.7326
[rel]
[ppm]

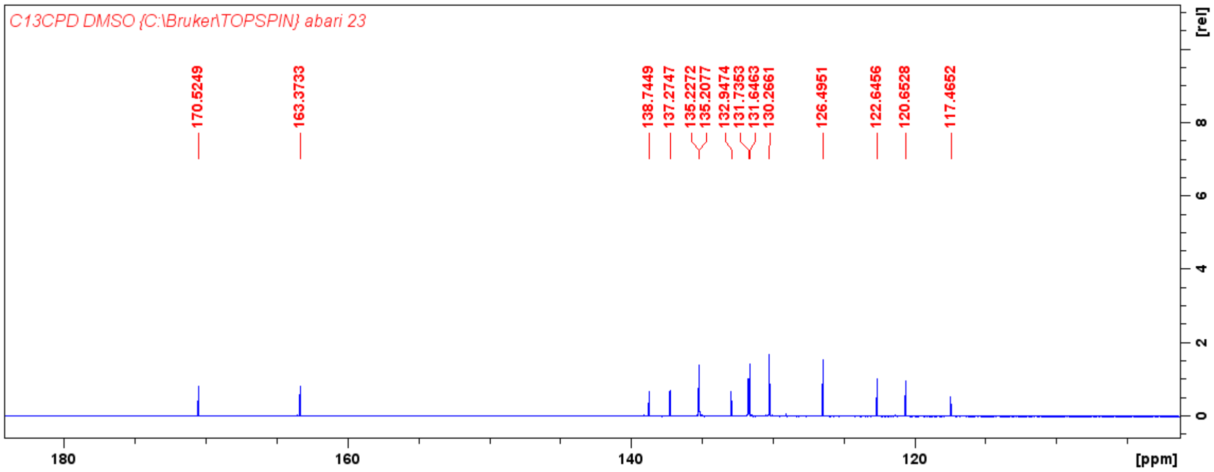
C13CPD DMSO {C:\Bruker\TOPSPIN} abari 23
170.5249
163.3733
138.7449
137.2747
135.2272
135.2077
132.9474
131.7353
131.6463
130.2661
126.4951
122.6456
120.6528
117.4652
[rel]
[ppm]

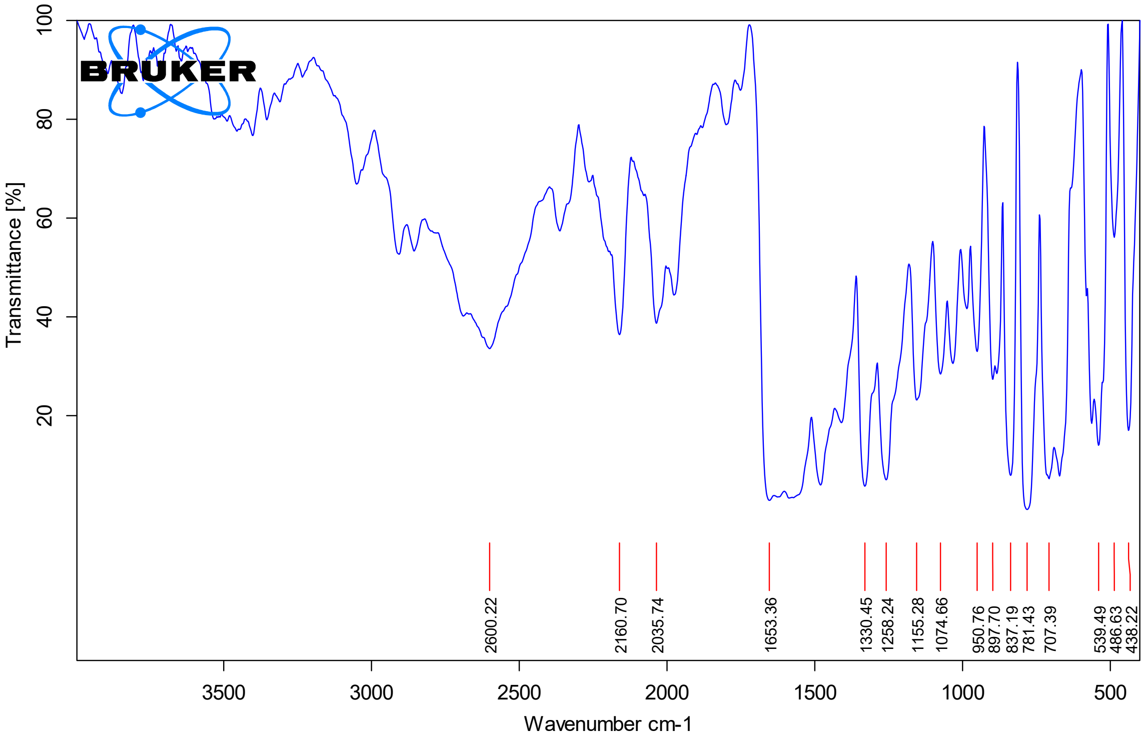

C:\Users\DELL\Documents\Bruker\OPUS_7.8.38\DATA\MEAS\Adel Elzab 9 NON 2020 Br-3.0	Adel Elzab 9 NON 2020 Br-3	Instrument	11/11/2020

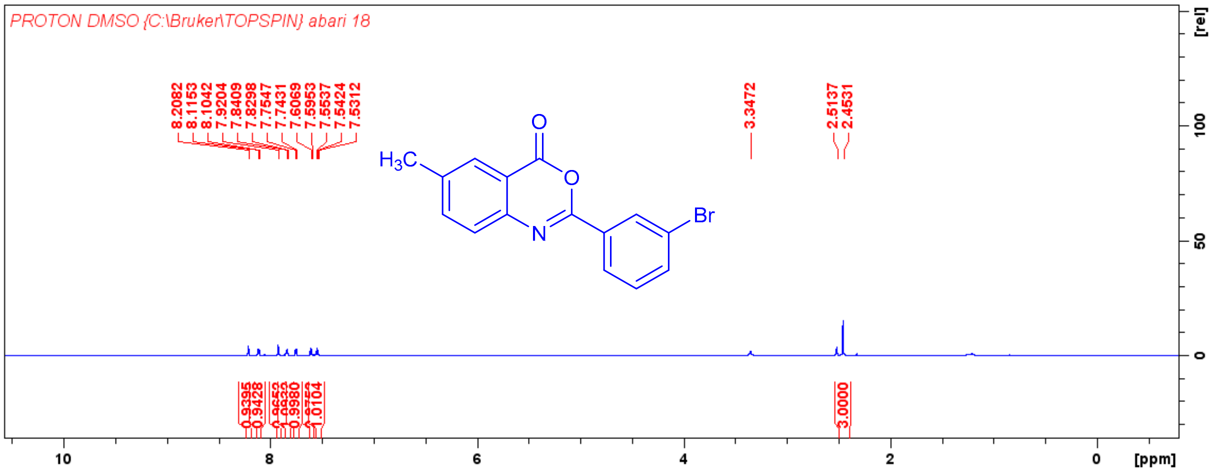
PROTON DMSO {C:\Bruker\TOPSPIN} abari 18
8.2082
8.1153
8.1042
7.9204
7.8409
7.8298
7.7547
7.7431
7.6069
7.5953
7.5537
7.5424
7.5312
3.3472
2.5137
2.4531
H_3C
O
O
N
Br
0.9395
0.9428
1.0104
3.0000
10
8
6
4
2
0
[ppm]
[rel]
100
50
0

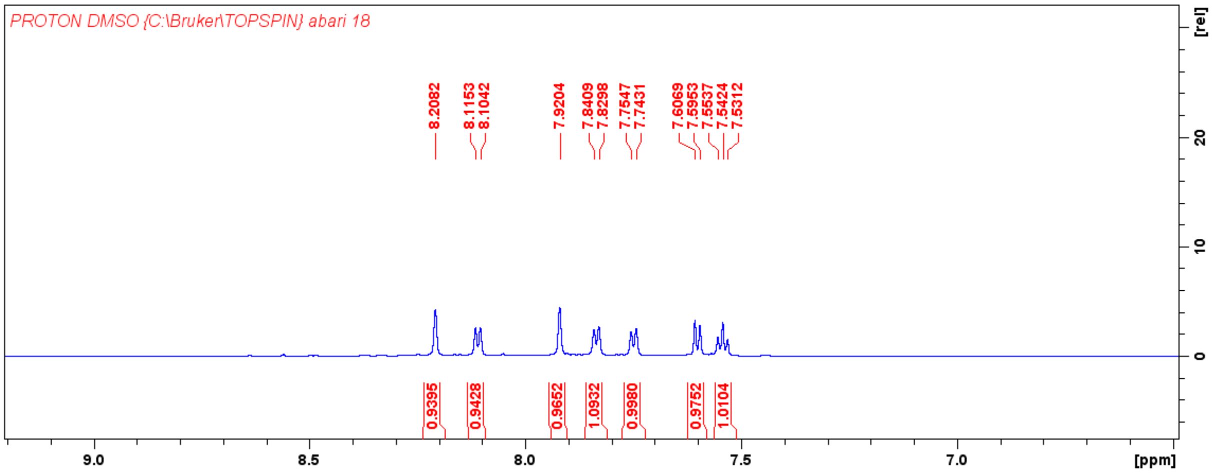
PROTON DMSO {C:\Bruker\TOPSPIN} abari 18
8.2082
8.1153
8.1042
7.9204
7.8409
7.8298
7.7547
7.7431
7.6069
7.5953
7.5537
7.5424
7.5312
0.9395
0.9428
0.9652
1.0932
0.9980
0.9752
1.0104
9.0
8.5
8.0
7.5
7.0
[ppm]
[rel]
20
10
0

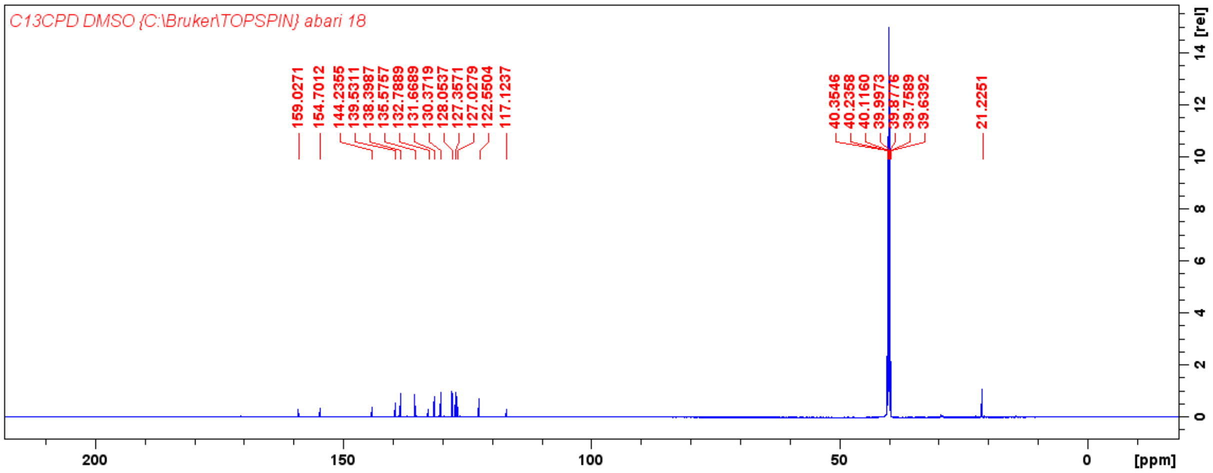
C13CPD DMSO {C:\Bruker\TOPSPIN} abari 18
159.0271
154.7012
144.2355
139.5311
138.3987
135.5757
132.7889
131.6689
130.3719
128.0537
127.3571
127.0279
122.5504
117.1237
40.3546
40.2358
40.1160
39.9973
39.8776
39.7589
39.6392
21.2251
200
150
100
50
0
[ppm]
[rel]
14
12
10
8
6
4
2
0

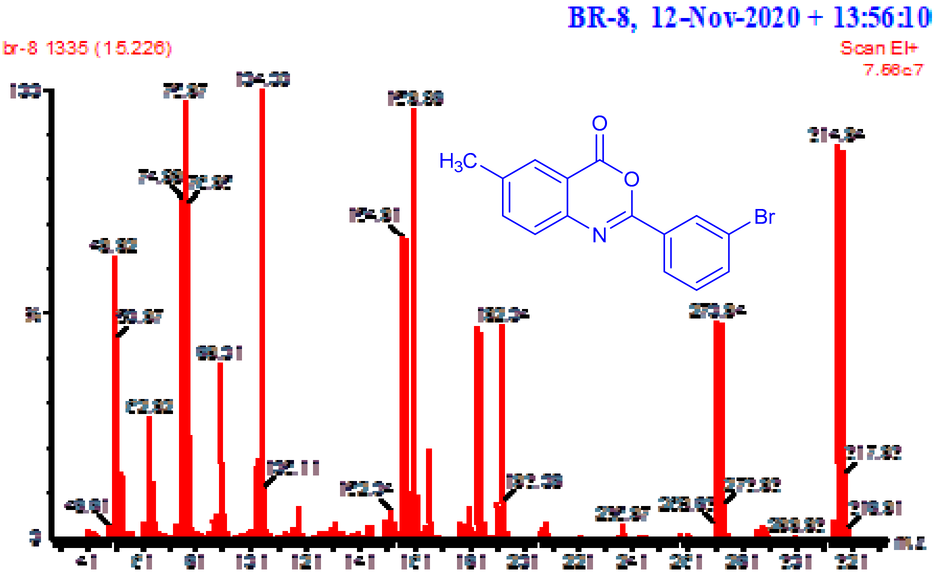

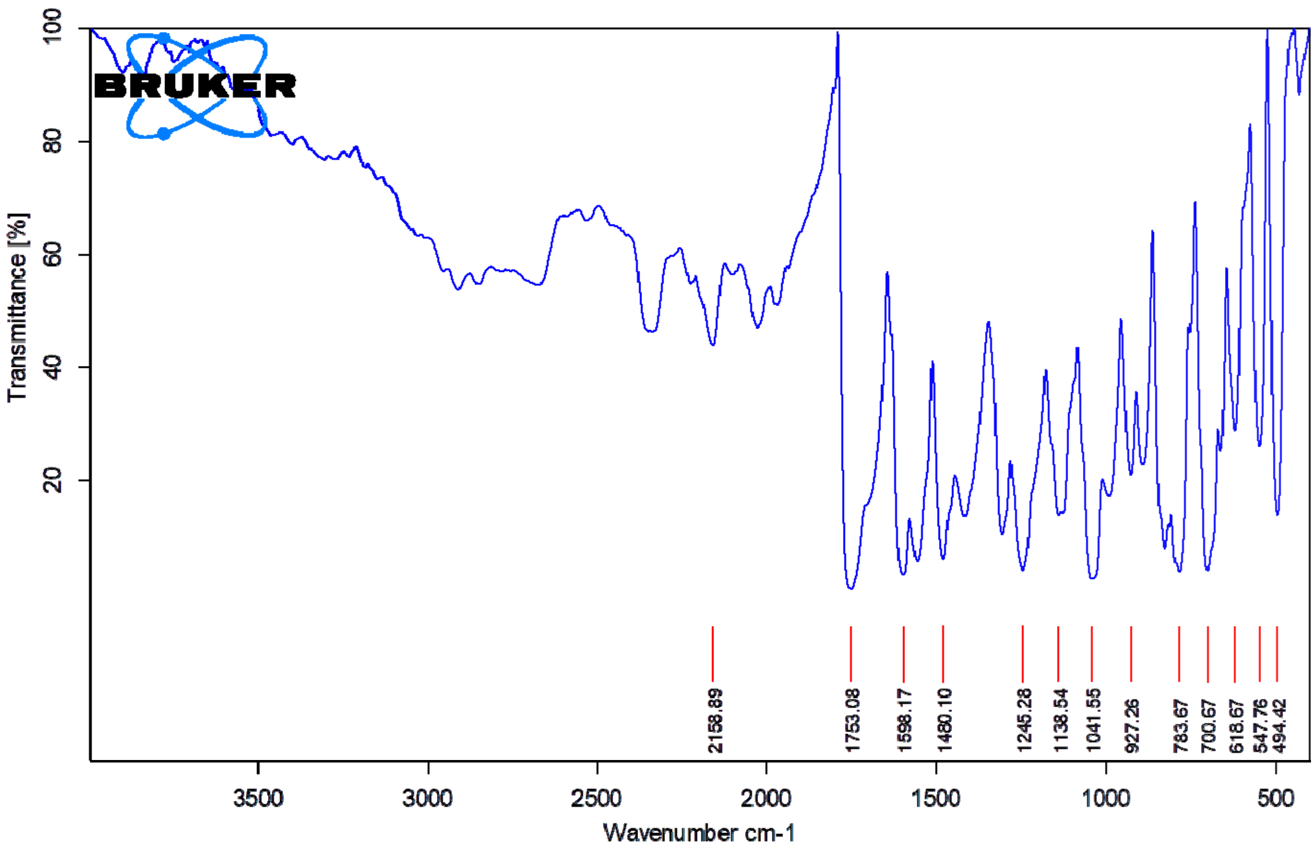

C:\Users\DELL\Documents\Bruker\OPUS_7.8.38\DATA\MEAS\Adel Elzab 9 NON 2020 Br-8.0 Adel Elzab 9 NON 2020 Br-8 Instrument	11/11/2020

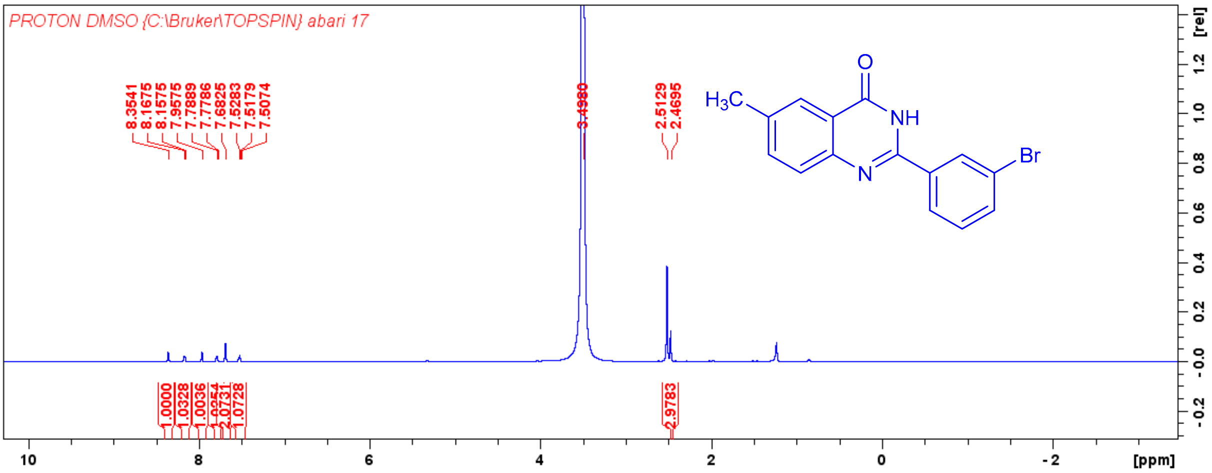
PROTON DMSO {C:\Bruker\TOPSPIN} abari 17
8.3541
8.1675
8.1575
7.9575
7.7889
7.7786
7.6825
7.5283
7.5179
7.5074
3.4980
2.5129
2.4695
H3C
O
NH
N
Br
1.0000
1.0328
1.0036
1.0254
2.0731
1.0728
2.9783
[ppm]
[rel]

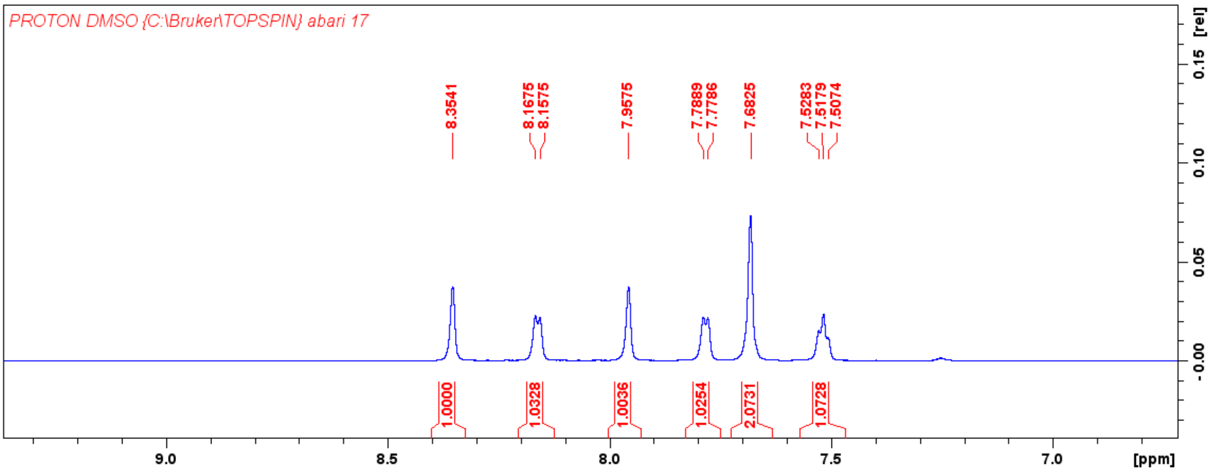
PROTON DMSO {C:\Bruker\TOPSPIN} abari 17
8.3541
8.1675
8.1575
7.9575
7.7889
7.7786
7.6825
7.5283
7.5179
7.5074
1.0000
1.0328
1.0036
1.0254
2.0731
1.0728
[ppm]
[rel]

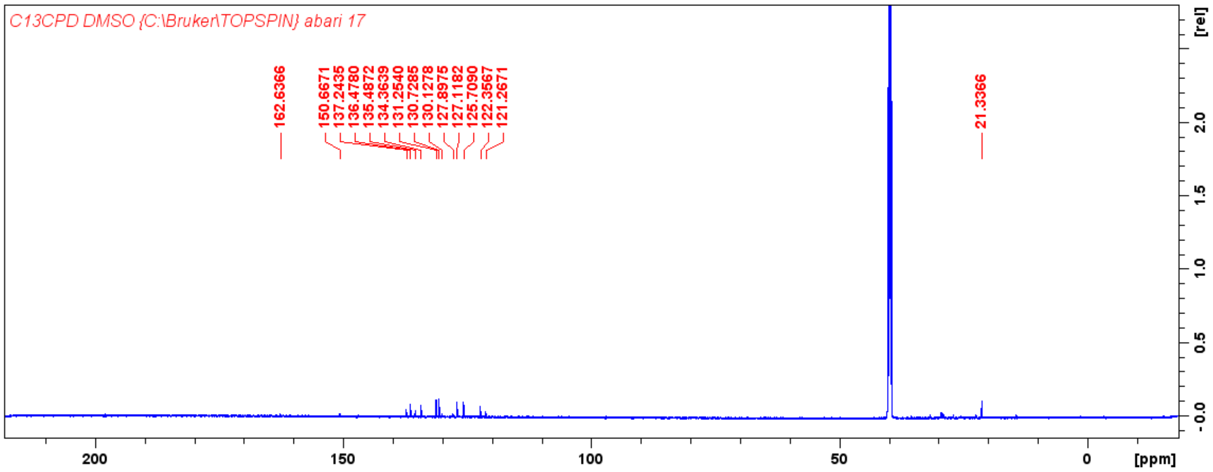
C13CPD DMSO {C:\Bruker\TOPSPIN} abari 17
162.6366
150.6671
137.2435
136.4780
135.4872
134.3639
131.2540
130.7285
130.1278
127.8975
127.1182
125.7090
122.3567
121.2671
21.3366
[rel]
[ppm]

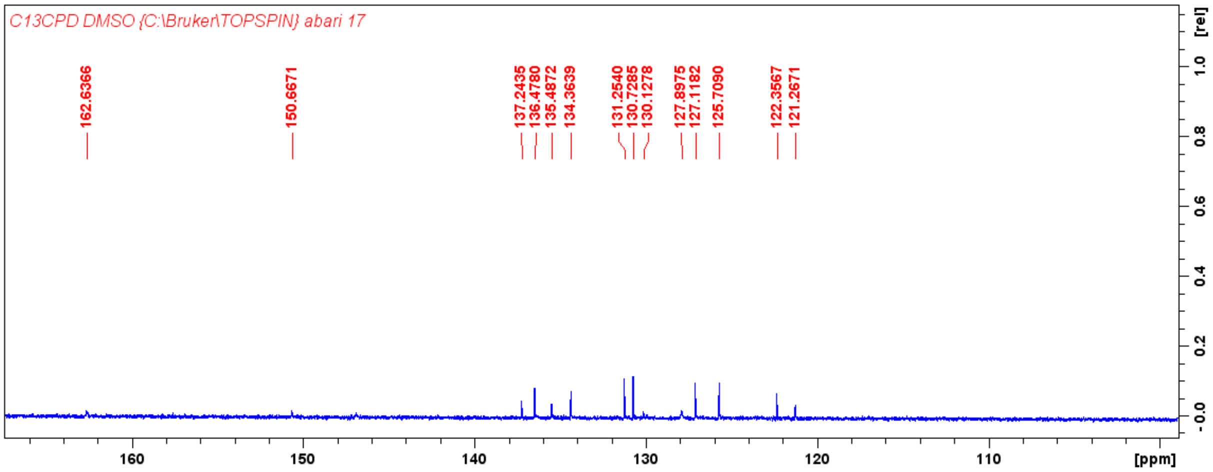
C13CPD DMSO {C:\Bruker\TOPSPIN} abari 17
162.6366
150.6671
137.2435
136.4780
135.4872
134.3639
131.2540
130.7285
130.1278
127.8975
127.1182
125.7090
122.3567
121.2671
[rel]
[ppm]

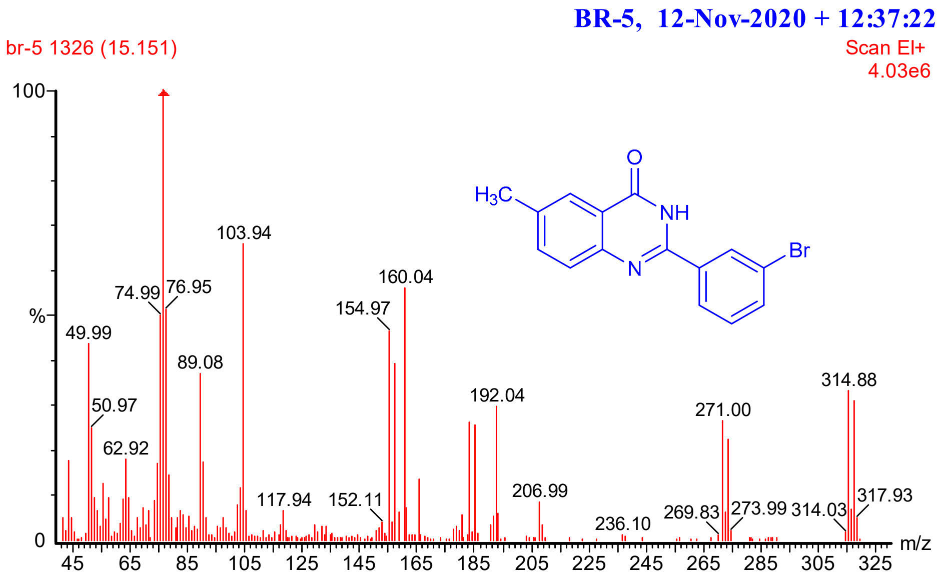

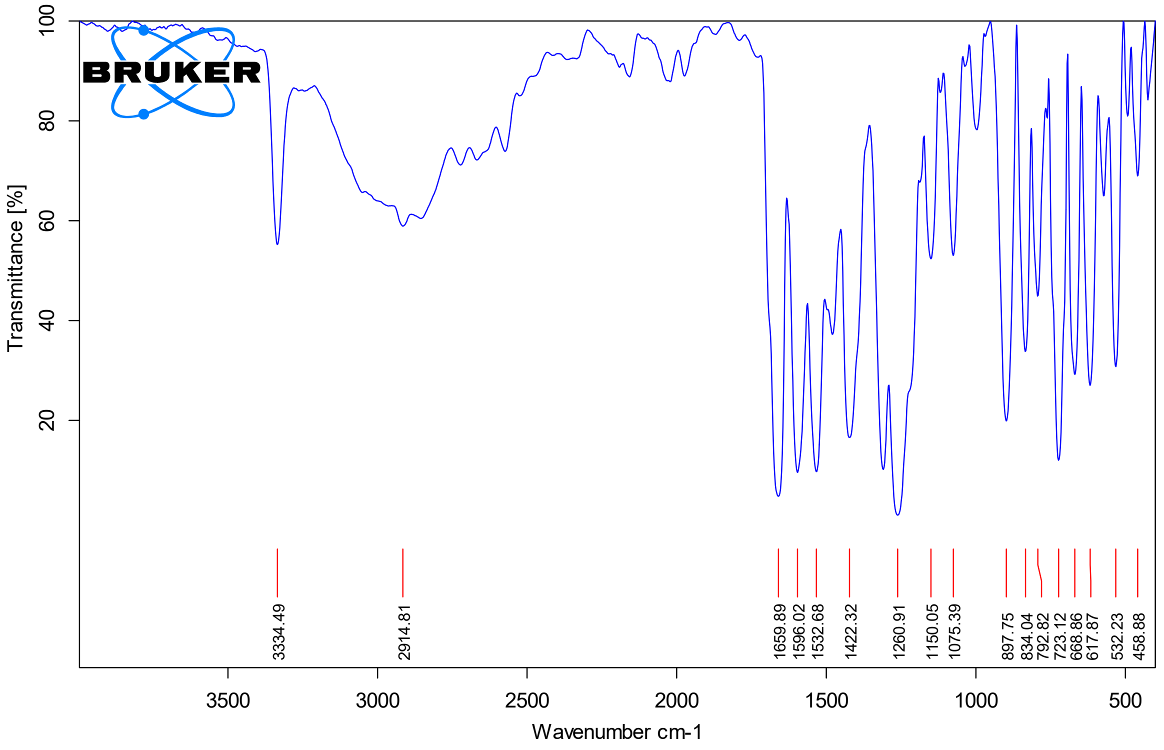

C:\Users\DELL\Documents\Bruker\OPUS_7.8.38\DATA\MEAS\Adel Elzab 9 NON 2020 Br-4.0	Adel Elzab 9 NON 2020 Br-4	Instrument	11/11/2020

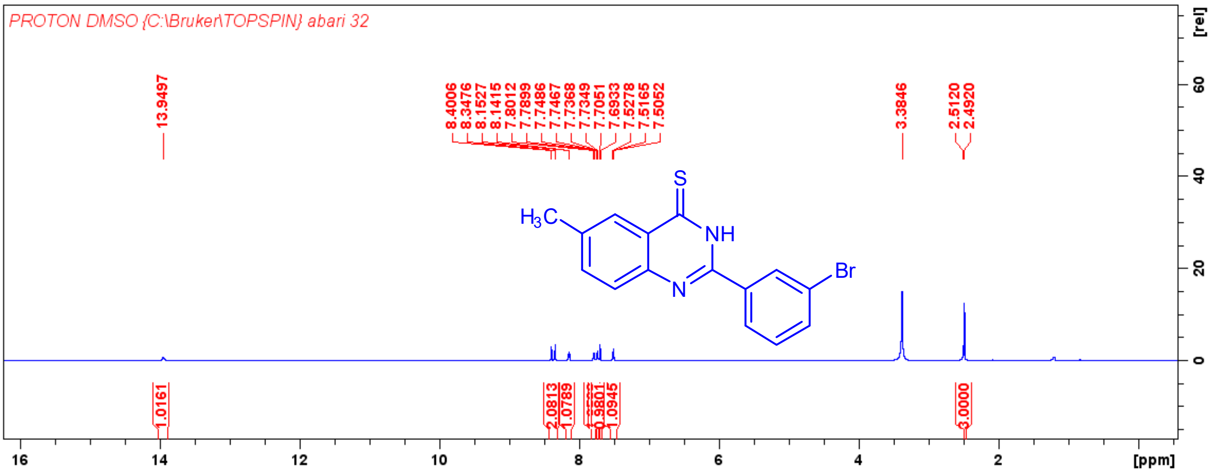
PROTON DMSO {C:\Bruker\TOPSPIN} abari 32
13.9497
8.4006
8.3476
8.1527
8.1415
7.8012
7.7899
7.7486
7.7467
7.7368
7.7349
7.7051
7.6933
7.5278
7.5165
7.5052
3.3846
2.5120
2.4920
S
NH
H3C
N
Br
1.0161
2.0813
1.0789
0.9801
1.0945
3.0000
[rel]
[ppm]

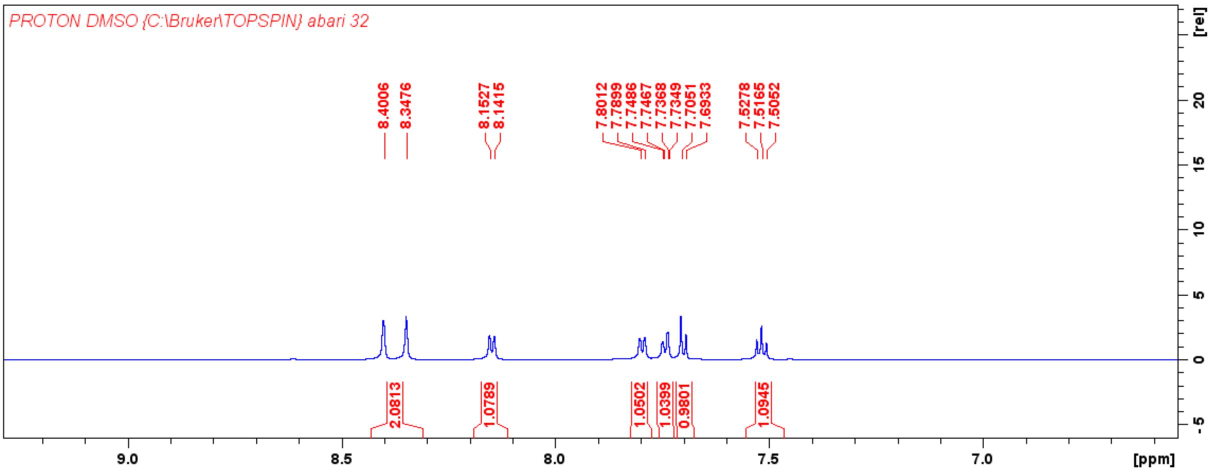
PROTON DMSO {C:\Bruker\TOPSPIN} abari 32
8.4006
8.3476
8.1527
8.1415
7.8012
7.7899
7.7486
7.7467
7.7368
7.7349
7.7051
7.6933
7.5278
7.5165
7.5052
2.0813
1.0789
1.0502
1.0399
0.9801
1.0945
[rel]
[ppm]

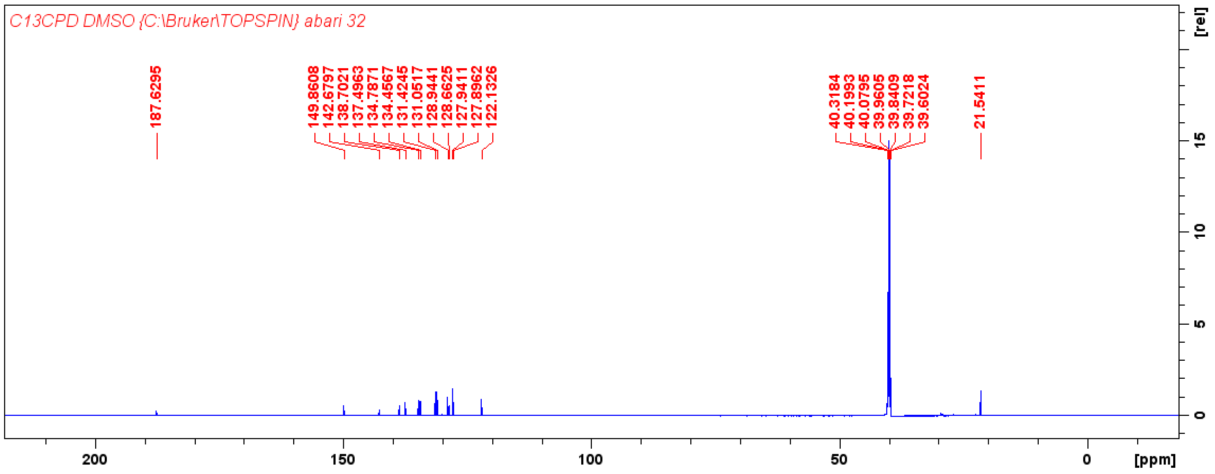
C13CPD DMSO {C:\Bruker\TOPSPIN} abari 32
187.6295
149.8608
142.6797
138.7021
137.4963
134.7871
134.4567
131.4245
131.0517
128.9441
128.6625
127.9411
127.8962
122.1326
40.3184
40.1993
40.0795
39.9605
39.8409
39.7218
39.6024
21.5411
[rel]
[ppm]

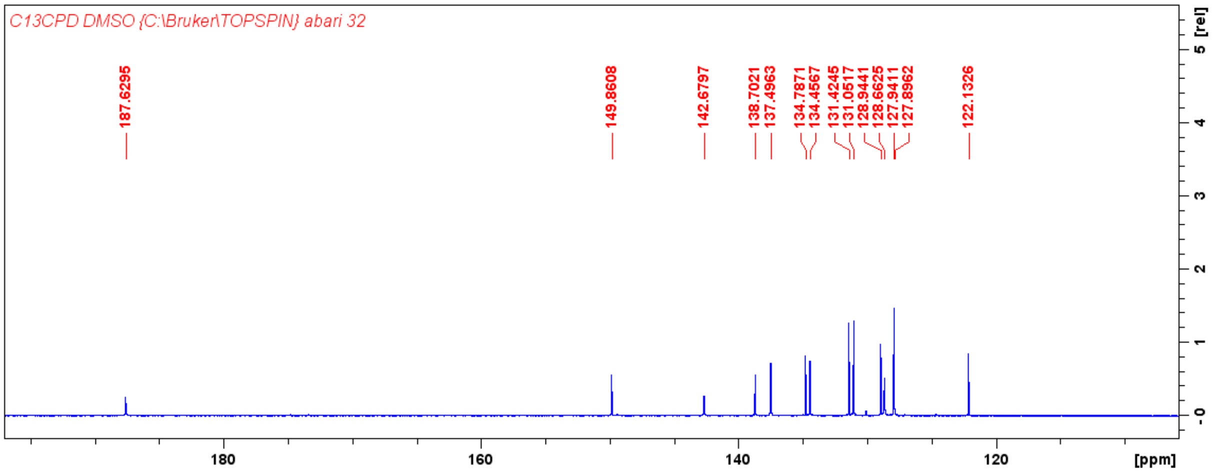
C13CPD DMSO {C:\Bruker\TOPSPIN} abari 32
187.6295
149.8608
142.6797
138.7021
137.4963
134.7871
134.4567
131.4245
131.0517
128.9441
128.6625
127.9411
127.8962
122.1326
[rel]
[ppm]

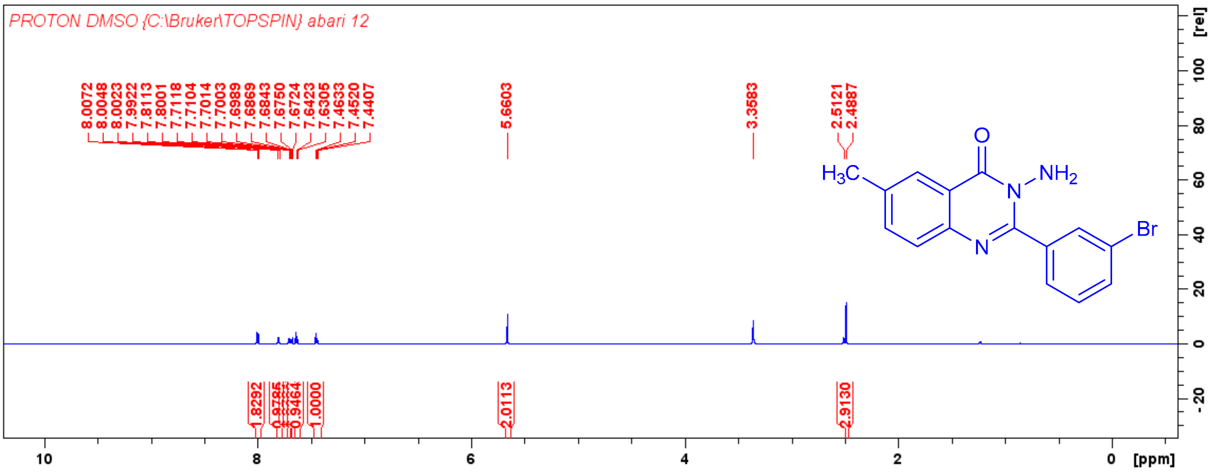
PROTON DMSO {C:\Bruker\TOPSPIN} abari 12
8.0072 8.0048 8.0023 7.9922 7.8113 7.8001 7.7118 7.7104 7.7014 7.7003 7.6989 7.6869 7.6843 7.6750 7.6724 7.6423 7.6305 7.4633 7.4520 7.4407
5.6603
3.3583
2.5121 2.4887
O
H_3C
NH_2
N
N
Br
1.8292
0.9464
1.0000
2.0113
2.9130
10 8 6 4 2 0 [ppm]
[rel]
100 80 60 40 20 0 -20

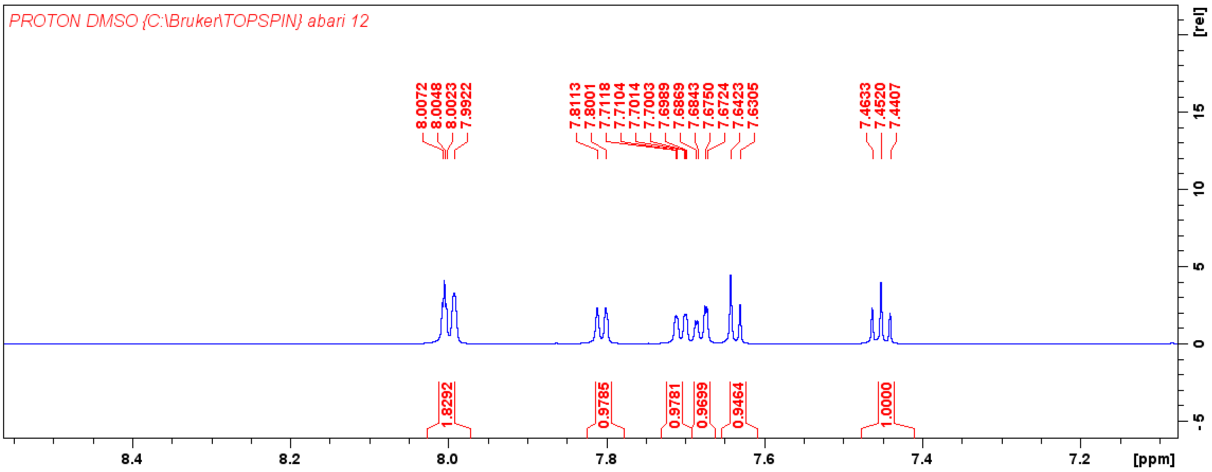
PROTON DMSO {C:\Bruker\TOPSPIN} abari 12
8.0072 8.0048 8.0023 7.9922
7.8113 7.8001 7.7118 7.7104 7.7014 7.7003 7.6989 7.6869 7.6843 7.6750 7.6724 7.6423 7.6305
7.4633 7.4520 7.4407
1.8292
0.9785
0.9781
0.9699
0.9464
1.0000
8.4 8.2 8.0 7.8 7.6 7.4 7.2 [ppm]
[rel]
15 10 5 0 -5

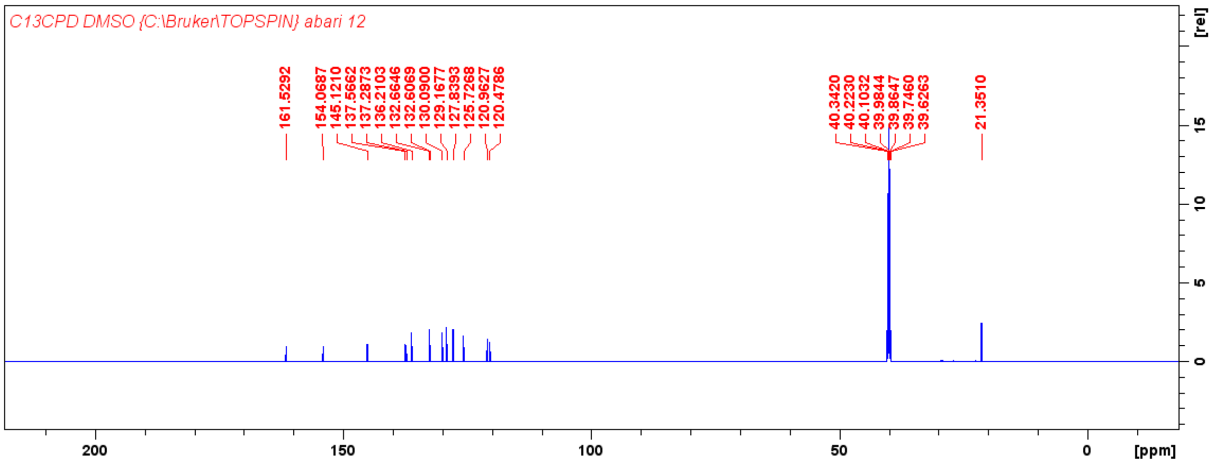
C13CPD DMSO {C:\Bruker\TOPSPIN} abari 12
161.5292
154.0687
145.1210
137.5662
137.2873
136.2103
132.6646
132.6069
130.0900
129.1677
127.8393
125.7268
120.9627
120.4786
40.3420
40.2230
40.1032
39.9844
39.8647
39.7460
39.6263
21.3510
[rel]
[ppm]

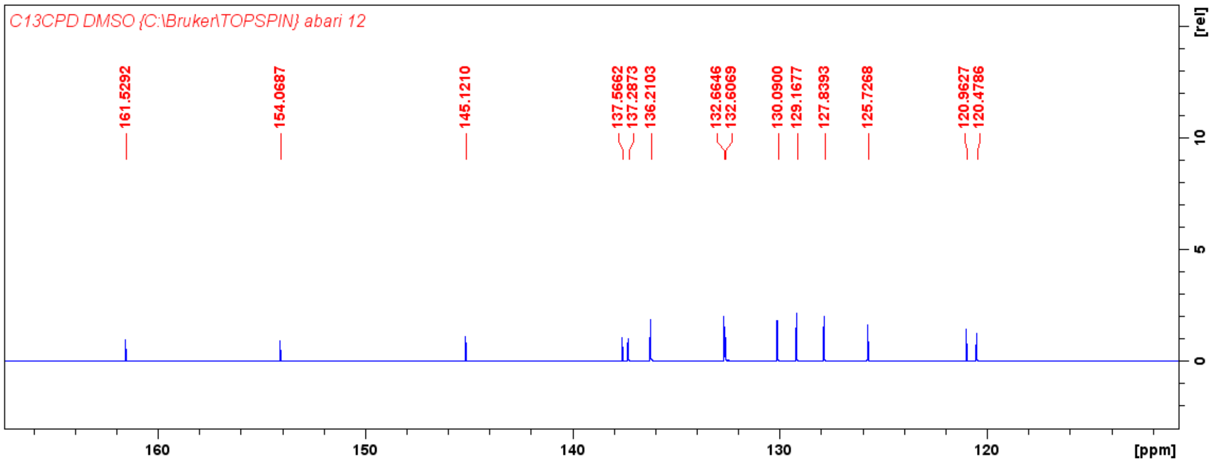
C13CPD DMSO {C:\Bruker\TOPSPIN} abari 12
161.5292
154.0687
145.1210
137.5662
137.2873
136.2103
132.6646
132.6069
130.0900
129.1677
127.8393
125.7268
120.9627
120.4786
[rel]
[ppm]

BR-2, 12-Nov-2020 + 09:58:54

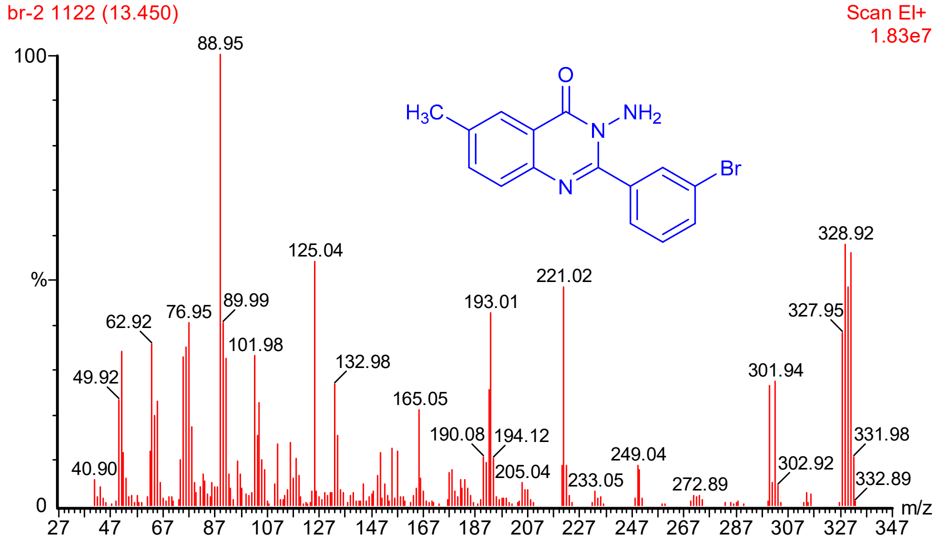

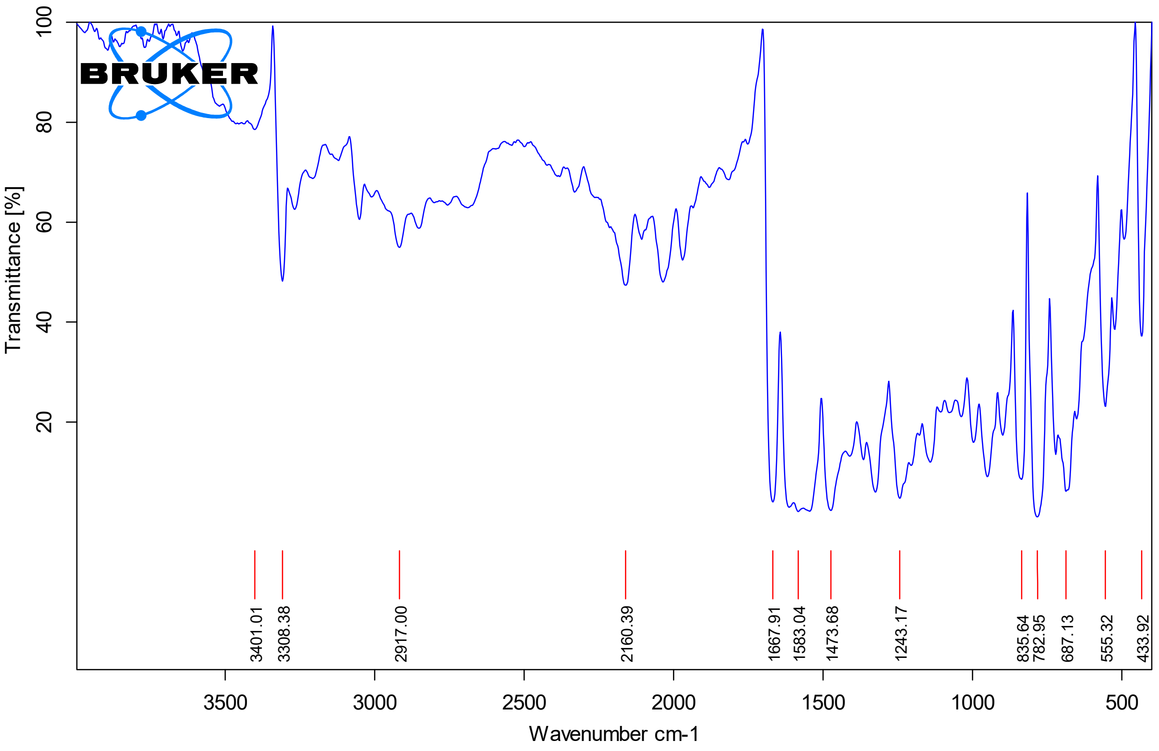

C:\Users\DELL\Documents\Bruker\OPUS_7.8.38\DATA\MEAS\Adel Elzab 9 NON 2020 Br-1.0 Adel Elzab 9 NON 2020 Br-1	Instrument	11/11/2020

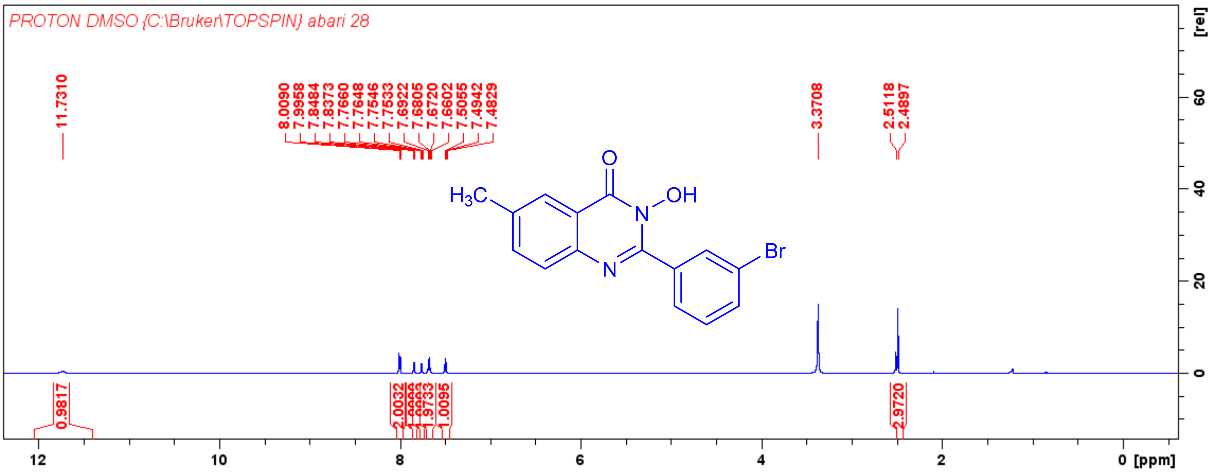
PROTON DMSO {C:\Bruker\TOPSPIN} abari 28
11.7310
8.0090
7.9958
7.8484
7.8373
7.7660
7.7648
7.7546
7.7533
7.6922
7.6805
7.6720
7.6602
7.5055
7.4942
7.4829
3.3708
2.5118
2.4897
O
N
OH
H3C
Br
N
0.9817
2.0032
1.0000
1.0003
1.9733
1.0095
2.9720
12
10
8
6
4
2
0 [ppm]
[rel]
60
40
20
0

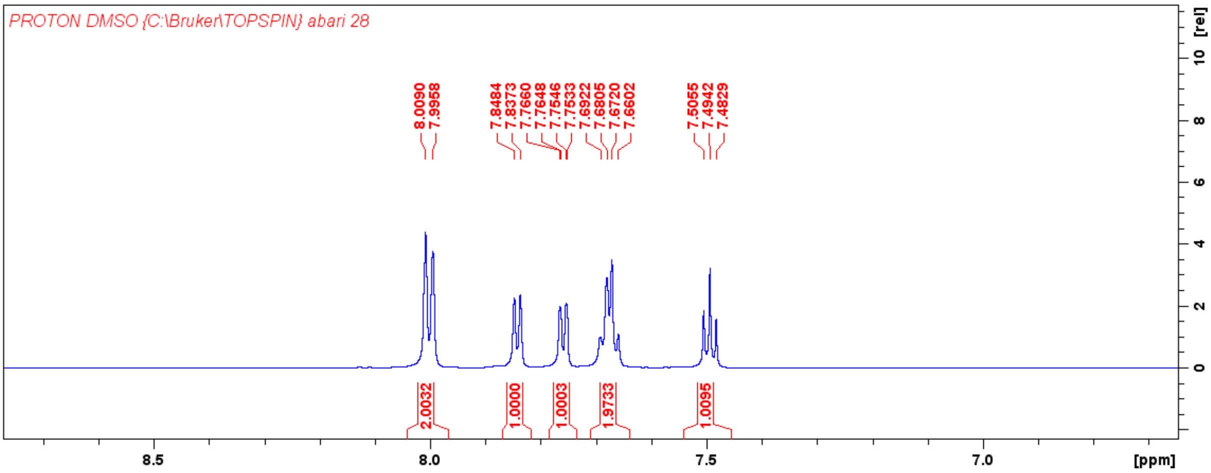
PROTON DMSO {C:\Bruker\TOPSPIN} abari 28
8.0090
7.9958
7.8484
7.8373
7.7660
7.7648
7.7546
7.7533
7.6922
7.6805
7.6720
7.6602
7.5055
7.4942
7.4829
2.0032
1.0000
1.0003
1.9733
1.0095
8.5
8.0
7.5
7.0
[ppm]
[rel]
10
8
6
4
2
0

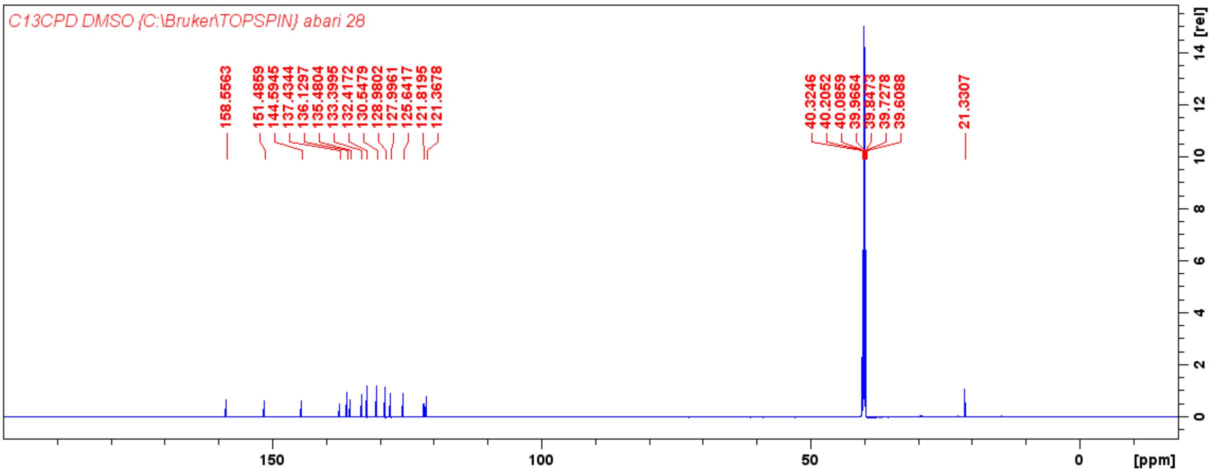
C13CPD DMSO {C:\Bruker\TOPSPIN} abari 28
158.5563
151.4859
144.5945
137.4344
136.1297
135.4804
133.3995
132.4172
130.5479
128.9802
127.9961
125.6417
121.8195
121.3678
40.3246
40.2052
40.0859
39.9664
39.8473
39.7278
39.6088
21.3307
150
100
50
0
[ppm]
[rel]
14
12
10
8
6
4
2
0

BR-3, 12-Nov-2020 + 11:53:56

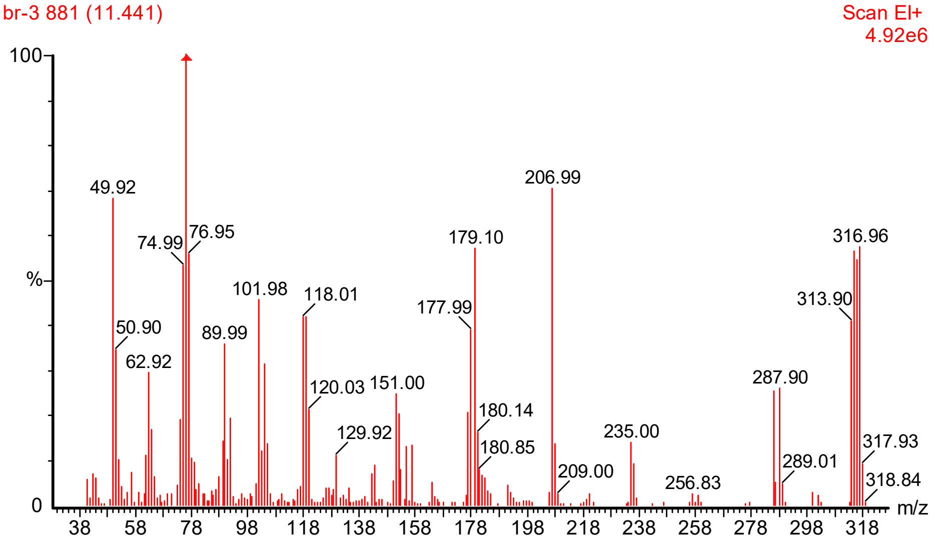

Supplement: Supplementary file 1 [file molecules-28-00120-s001.zip › molecules-2056740-supplementary.pdf]
